# Reciprocal Interactions Between Human GV-Oocytes and Cumulus Cells: Effects on GVBD, ROS Production, and AMPK Expression

**DOI:** 10.3390/medicina61122107

**Published:** 2025-11-26

**Authors:** Zhaoqiao Ban, Plamen Todorov, Gohar Rahimi, Christine Skala, Volodimir Isachenko

**Affiliations:** 1 Department of Obstetrics and Gynecology, Medical Faculty, Cologne University, 50931 Cologne, Germany; banzhaoqiao@gmail.com (Z.B.); christine.skala@uk-koeln.de (C.S.); 2 Institute of Biology and Immunology of Reproduction of Bulgarian Academy of Sciences (BAS), 1113 Sofia, Bulgaria; plamen.ivf@gmail.com; 3 Medizinisches Versorgungszentrum AMEDES für IVF-und Pränatalmedizin in Köln GmbH, 50968 Cologne, Germany

**Keywords:** oocyte maturation, germinal vesicle breakdown (GVBD), cumulus cells, co-culture, oxidative stress, AMPK, in vitro maturation, reactive oxygen species, assisted reproductive technologies

## Abstract

*Background and Objectives:* The quality of cumulus cells (CCs) is a major determinant of germinal vesicle (GV) oocyte maturation, yet the reciprocal effect of GV oocytes on cumulus cell function remains unclear. *Materials and Methods:* GV oocytes were cultured with or without cumulus cells (only oocytes or Oocytes–CCs), and GVBD rates were evaluated after 24 h. In parallel, cumulus cells were cultured either alone (only cumulus) or with oocytes (CCs + Oocytes). Cell morphology, growth, intracellular reactive oxygen species (ROS), and AMP-activated protein kinase (AMPK) expression were assessed by fluorescence and immunocytochemistry. *Results:* GVBD rates were significantly higher in Oocytes + CCs than in only oocytes (66.7% vs. 18.2%, *p* < 0.05). Cumulus cells co-cultured with oocytes exhibited improved growth, tighter cell connections, and greater extracellular matrix formation. ROS levels were reduced in CCs + Oocytes compared with only the cumulus group (12.1% vs. 21.9%, *p* < 0.01), whereas AMPK expression increased markedly (229% of CCs–Oocytes, *p* < 0.0001). *Conclusions:* In vitro co-culture underscores not only the supportive role of cumulus cells in oocyte maturation but also a reciprocal, beneficial effect of oocytes on cumulus cell viability and function, revealing the bidirectional nature of oocyte–cumulus interactions.

## 1. Introduction

In vitro maturation (IVM) of oocytes has emerged as a cornerstone in assisted reproductive technologies (ART), offering a valuable alternative for patients who are unable or unwilling to undergo conventional ovarian stimulation protocols [1,2]. This approach is particularly advantageous for individuals with specific clinical conditions, such as polycystic ovary syndrome (PCOS) [3,4], where ovarian hyperstimulation poses significant risks [5,6,7]. By circumventing the need for extensive hormonal stimulation, IVM provides a safer and more cost-effective solution for fertility preservation and treatment [8]. Despite these advantages, the widespread application of IVM in clinical practice remains limited by suboptimal maturation rates and inconsistent outcomes.

One of the primary challenges in optimizing IVM lies in the intricate interplay between oocytes and their surrounding cumulus cells (CCs), which form an essential component of the oocyte microenvironment. Cumulus cells are tightly associated with oocytes [9,10], providing metabolic support, regulating oxidative stress, and facilitating intracellular communication through gap junctions. These interactions are critical for oocyte growth, development, and maturation. However, the delicate balance within this microenvironment is often disrupted during in vitro culture, leading to suboptimal maturation outcomes. An essential milestone of oocyte nuclear maturation is germinal vesicle breakdown (GVBD), which marks the resumption of meiosis and the transition from the prophase I arrest to subsequent maturation stages. GVBD represents the irreversible commitment of the oocyte to mature, and is widely used as an early indicator of oocyte competence during in vitro maturation (IVM). In human IVM models, especially when complete progression to the metaphase II (MII) stage may be limited, GVBD occurrence serves as a reliable proxy for assessing the activation of oocyte maturation pathways [11].

Oxidative stress is a major impediment to successful IVM [12], driven by the overproduction of reactive oxygen species (ROS) under in vitro conditions. Excessive ROS can induce cellular damage, compromise mitochondrial function, and impair both oocyte and cumulus cell viability [13]. While the physiological presence of ROS is necessary for signaling pathways involved in maturation, maintaining ROS levels within a narrow range is crucial for ensuring oocyte competence. Cumulus cells play a vital role in this process by scavenging excess ROS and modulating the oxidative environment around the oocyte [14]. However, their capacity to mitigate oxidative stress during IVM is often diminished, leading to a hostile microenvironment that undermines maturation efficiency.

In this context, AMP-activated protein kinase (AMPK) has garnered attention as a key regulator of cellular energy homeostasis and oxidative stress responses. AMPK is a serine/threonine kinase that becomes activated under conditions of low energy availability or oxidative stress, promoting metabolic adaptations that enhance cellular resilience [15,16,17]. In oocytes, AMPK activation has been linked to improved developmental competence, increased mitochondrial activity, and enhanced resistance to oxidative damage. Similarly, AMPK activation in cumulus cells may play a pivotal role in maintaining the metabolic and oxidative balance necessary for supporting oocyte maturation [17]. Despite these potential benefits, the role of AMPK in the cumulus cell–oocyte interaction during IVM remains largely unexplored.

The co-culture of cumulus cells with oocytes represents a promising strategy to overcome the limitations of traditional IVM protocols [18]. By preserving the natural cellular interactions that occur in vivo, co-culture systems can enhance the microenvironment surrounding the oocyte, improving both its metabolic state and oxidative stability [19].

Preliminary studies have suggested that co-culturing cumulus cells with oocytes not only enhances oocyte maturation rates but also modulates key cellular pathways, including those associated with ROS management and AMPK activation [20]. However, a comprehensive understanding of these mechanisms is lacking, and the relative contributions of cumulus cell-mediated ROS regulation and AMPK activation to oocyte maturation efficiency have yet to be fully elucidated.

This study aims to address these gaps by investigating the effects of cumulus cell–oocyte co-culture on IVM outcomes, focusing on GVBD occurrence as well as changes in cumulus cell morphology, intracellular ROS levels, and AMPK protein expression. Specifically, we hypothesize that co-culture conditions will create a more favorable microenvironment for oocyte development by reducing oxidative stress and enhancing energy metabolism. Through detailed analysis of these interactions, this research seeks to provide novel insights into the molecular mechanisms underlying oocyte maturation and to propose refinements to IVM protocols that could improve their clinical utility. By bridging the knowledge gap between oxidative stress regulation, AMPK activation, and cumulus cell–oocyte interactions, this study contributes to the broader goal of optimizing ART outcomes. These findings have the potential to inform not only IVM practices but also other ART techniques where oxidative stress and cellular energy balance are critical determinants of success.

In fact, the quality of cumulus cells is a key determinant of the efficiency of germinal vesicle-oocyte maturation. Nevertheless, evidence regarding the reciprocal influence of GV-oocytes on cumulus cell function remains scarce.

The reciprocal metabolic and redox interactions between oocytes and cumulus cells are not only of fundamental biological interest but also hold considerable translational potential in reproductive and general regenerative medicine. In the clinical context of assisted reproduction, understanding the mechanisms that govern oocyte–cumulus cell communication may lead to optimized in vitro maturation (IVM) systems capable of enhancing oocyte competence and pregnancy outcomes in patients with infertility, particularly those affected by polycystic ovary syndrome (PCOS), premature ovarian insufficiency, or advanced maternal age. Beyond reproductive medicine, the regulatory role of AMP-activated protein kinase (AMPK) and redox homeostasis highlighted in this study reflects universal mechanisms of cellular stress resistance, energy adaptation, and metabolic integrity. Insights gained from these oocyte–cumulus models may therefore inform broader therapeutic strategies targeting oxidative stress–related pathologies, such as metabolic syndrome, diabetes, neurodegeneration, and ischemic injury. This dual relevance underscores the bridge between reproductive biology and systemic cell physiology, positioning oocyte–cumulus communication as a model for studying cell–cell cooperation under oxidative stress.

In the present study, we aimed to examine not only the effect of cumulus cells on the dynamics of GV-oocyte germinal vesicle breakdown (GVBD), but also the impact of the presence or absence of oocytes on the quality of cultured cumulus cells. By shifting the perspective from the well-established role of cumulus cells in supporting oocyte maturation to the less explored question of how oocytes may modulate cumulus cell physiology, this work provides new insights into the bidirectional nature of oocyte–cumulus interactions.

## 2. Materials and Methods

### 2.1. Ethical Standards

The authors assert that all procedures contributing to this work comply with the ethical standards of the relevant national and institutional committees on human experimentation and with the Helsinki Declaration of 1975, as revised in 2008. Immature GV oocytes and cumulus cells were obtained from 23 patients undergoing ovarian tissue cryopreservation at the Women’s Clinic, University of Cologne, Germany.

Unless otherwise specified, all chemicals were obtained from Sigma Chemical Co. (St. Louis, MO, USA).

### 2.2. Sample Collection After Follicle Puncture

Immature germinal vesicle (GV) oocytes and cumulus cells were obtained from 23 patients undergoing ovarian tissue cryopreservation at the Women’s Clinic, University of Cologne, Germany. All participants provided informed consent before the procedure. 

Oocyte–cumulus complexes were obtained during the preparation of ovarian tissue for cryopreservation through three procedural steps:

1. The cortical layer was separated from the medulla.
2. Visible follicles within the cortical layer were mechanically disrupted.
3. The large ovarian fragment was dissected into smaller pieces prior to freezing.

### 2.3. Isolation of Cumulus Cells and GV-Oocytes

Immediately after puncture, follicular fluid was transferred into sterile tubes and processed under aseptic conditions in a laminar flow hood. Oocyte–cumulus complexes (OCCs) were retrieved and washed three times in Multipurpose Handling Medium (Fujifilm, Irvine Scientific, Santa Ana, CA, USA). The OCCs were then cultured for 3 h in Continuous Single Culture Complete (CSCM-C) medium (Fujifilm Irvine Scientific, Santa Ana, CA, USA) under light mineral oil (Fujifilm Irvine Scientific, Santa Ana, CA, USA) in a CO_2_-incubator until enzymatic treatment. Removal of cumulus cells was performed using 60 IU/mL hyaluronidase (Fujifilm Irvine Scientific, Santa Ana, CA, USA), followed by three washes in CSCM-C. All oocytes that remained at GV stage were used for in vitro maturation. The removed cumulus cells were collected by two centrifugations in DMEM/F-12 medium (Gibco, Thermo Fisher Scientific, Waltham, MA, USA) supplemented with 10% fetal bovine serum (FBS) and 1% penicillin–streptomycin at 360 g, and the pellet was resuspended in fresh medium.

### 2.4. In Vitro Maturation of GV-Oocytes

GV-oocytes were cultured in DMEM/F-12 medium supplemented with 10% FBS and 1% penicillin–streptomycin. Oocytes were randomly assigned to two groups: (1) GV-oocytes cultured without cumulus cells (GV-oocytes-CCs, *n* = 11), and (2) GV-oocytes cultured with cumulus cells (GV-oocytes + CCs, *n* = 12). In the group GV-oocytes + CCs, GV-oocytes were placed directly onto the CC layer in the same well (no separation by membrane/insert), and cultures proceeded without medium change for 24 h. Cells of both groups were incubated at 37 °C in a humidified atmosphere containing 5% CO_2_. After 24 h of culture, nuclear status was assessed by inverted microscopy. Oocytes with a visible germinal vesicle (GV) were classified as “GV intact,” whereas those without a GV were classified as “GVBD occurred.” Fisher’s Exact Test (*n* = 23, small sample size) was used to compare GVBD rates between groups.

### 2.5. In Vitro Culture and Morphological Observation of Cumulus Cells

Cumulus cells were seeded into 10 mm culture dishes with 3 mL of DMEM/F-12 medium supplemented with 10% FBS and 1% penicillin–streptomycin and divided into two groups: (1) CCs cultured separately without oocytes (CCs-oocytes), and (2) CCs co-cultured with oocytes (CCs + Oocytes), in the CC + Oocyte group, oocytes and cumulus cells were maintained in direct physical contact in the same well (no membrane or insert), and the medium was not changed during the 24 h incubation to preserve paracrine signaling. Cultures were maintained at 37 °C in 5% CO_2_. Morphological characteristics, including cell size, shape, and degree of expansion, were documented at 24 h and 48 h using inverted microscopy at ×40 magnification.

### 2.6. Measurement of Intracellular ROS Levels

Intracellular ROS in cumulus cells was detected using H_2_DCFDA (2′,7′-dichlorodihydrofluorescein diacetate; Sigma-Aldrich, Darmstadt, Germany). Experimental groups were as described in Section 2.5, with an additional Positive Control group treated with 0.5 mmol/L H_2_O_2_ for 30 min to induce oxidative stress [21,22]. Cumulus cells were incubated with 10 μmol/L H_2_DCFDA in serum-free medium for 30 min at 37 °C in the dark. Cells were then washed three times with serum-free medium. Fluorescence signals were detected using a confocal microscope (LSM 700; Zeiss, Jena, Germany) with excitation/emission at 488/525 nm. Images were captured with consistent settings, and fluorescence intensity was quantified using ImageJ software (Version 1.47; NIH, Bethesda, MD, USA). Statistical analysis was performed using SPSS (Version 28.0; IBM Corp., Armonk, NY, USA) and GraphPad Prism (Version 9.0; GraphPad Software, San Diego, CA, USA). One-way ANOVA was used, followed by Tukey’s HSD post hoc test. A *p*-value < 0.05 was considered statistically significant.

### 2.7. Immunocytochemical Staining of Cumulus Cells

Cells of experimental groups included an additional Negative Control (no primary antibody) to confirm staining specificity. Cumulus cells were fixed with 4% paraformaldehyde at room temperature for 30 min, then permeabilized with 0.5% Triton X-100 in culture medium for 20 min. After blocking with phosphate-buffered saline (PBS) containing 1% bovine serum albumin (BSA) for 1 h, cells were incubated with anti-AMPK alpha 1 primary antibody(Abcam cat. no. ab32047 [Y365]; dilution 1:250) for 4 h at room temperature (Negative Control received no primary antibody). After three PBS washes, cells were incubated for 1 h with Alexa Fluor™ 350–conjugated goat anti-rabbit IgG (H + L) secondary antibody (Invitrogen, Thermo Fisher Scientific, A-11046, Waltham, MA, USA). AMPK expression was visualized by confocal microscopy (LSM 700; Zeiss, Jena, Germany), and fluorescence intensity was quantified with ImageJ software.

## 3. Results

### 3.1. GVBD Occurrence Under Different Culture Conditions

No statistically significant differences were observed between oocytes of both groups (patients whose oocytes were assigned to the Oocyte-CC group and the Oocyte + CC group with respect to these variables (*t*-test, *p* > 0.1).

**Figure 1:**
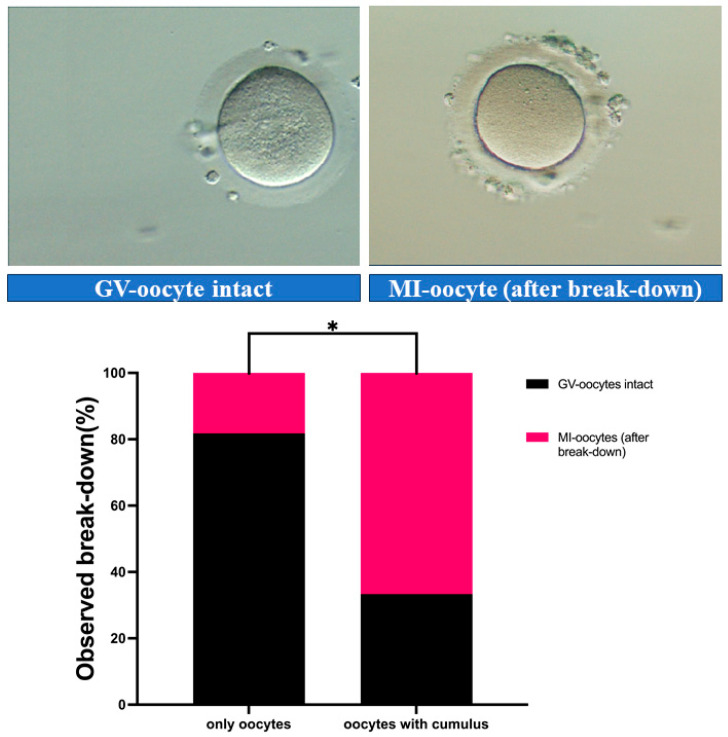
In vitro maturation of GV-oocytes. (**Left**) Intact GV-oocyte with a clearly visible germinal vesicle. (**Right**) GVBD occurred, as evidenced by the absence of a visible GV and homogeneous cytoplasm. (**Below**) Positive effect of presence of cumulus cells in culture medium on the rate of maturation to MI stage. * *p* < 0.01. Abbreviations: GVBD, germinal vesicle breakdown, metaphase I (MI).

Representative images of oocytes at different nuclear statuses after 24 h of culture are shown in Figure 1. Oocytes with a clearly visible germinal vesicle (GV) were classified as intact, while oocytes without a visible GV, typically exhibiting a homogeneous cytoplasm, were classified as MI oocytes (after breakdown). In oocytes of the Oocyte-CC group, 18% of oocytes underwent GVBD. In contrast, after co-culture with cumulus cells (Oocyte + CC group), 67% of oocytes underwent GVBD. Statistical analysis using Fisher’s Exact Test revealed a significant difference between maturation rate of oocytes in both groups (*p* < 0.04; Figure 1; Table 1).

### 3.2. Morphology and Growth Status of Cumulus Cells Cultured Under Different Culture Conditions

**Figure 2:**
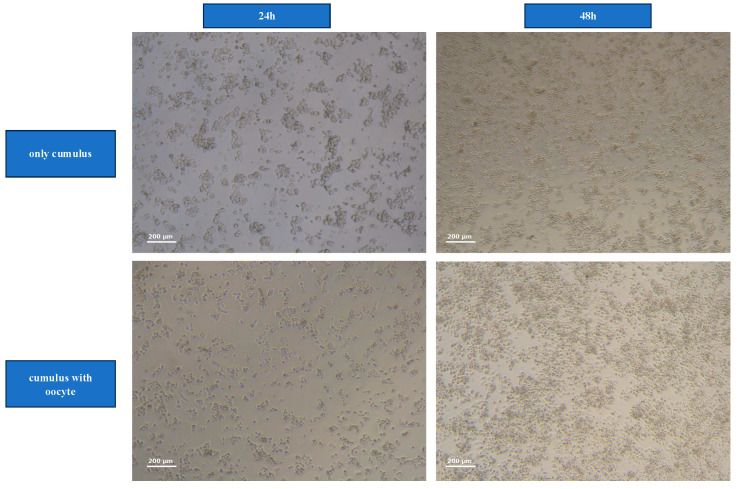
Morphological observation of cumulus cells (CCs) at 24 and 48 h cultured under different conditions.

The morphology and growth patterns of cumulus cells differed markedly between cells in group CCs + Oocytes and group CCs-Oocytes, as observed at 24 and 48 h of in vitro culture. Cumulus cells in the CC-Oocyte group exhibited isolated growth patterns, with slender morphology, limited expansion, and sparse intercellular connections. In contrast, cumulus cells in CC + Oocyte group displayed pronounced expansion, increased extracellular matrix production, and tighter cell–cell connections (Figure 2).

### 3.3. Intracellular ROS Levels in Cumulus Cells Cultured Under Different Culture Conditions

**Figure 3:**
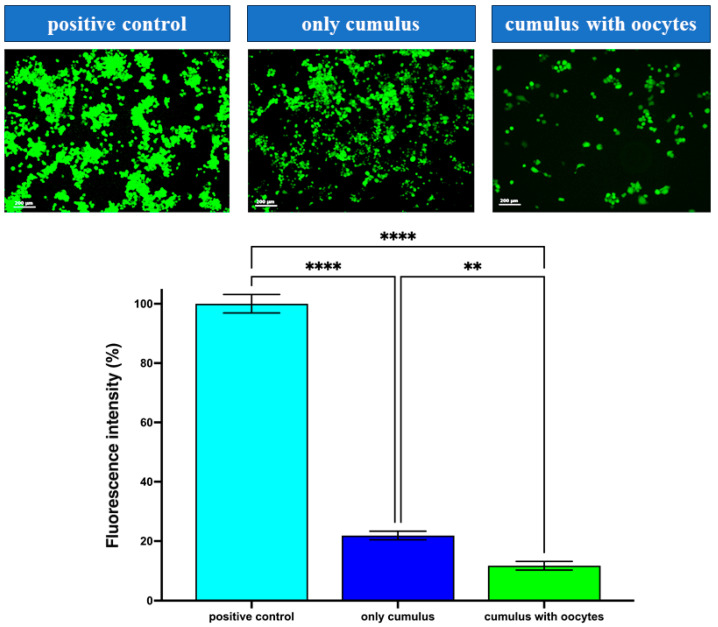
Reactive oxygen species (ROS) levels in cumulus cells cultured under different conditions. Representative images showing ROS fluorescence for the three conditions from left to right: positive control, cumulus without oocytes (group only cumulus), and cumulus co-cultured with oocytes (group CCs with Oocytes). (Below) The bar chart shows relative ROS fluorescence intensity for the CCs of three groups. ** *p* < 0.01, **** *p* < 0.0001. Abbreviations: ROS, reactive oxygen species.

Oxidative stress, as indicated by intracellular ROS levels, was significantly influenced by the culture conditions of cumulus cells. Quantification of fluorescence intensity revealed that the ROS levels in the only cumulus group were 21.87% of those in the Positive Control (H_2_O_2_-treated CCs) group, while ROS levels in the CC with Oocyte group were significantly lower at 12.1% of the Positive Control group (*p* < 0.01). These results demonstrate that co-culturing cumulus cells with oocytes effectively reduces intracellular ROS levels, thereby alleviating oxidative stress (Figure 3).

### 3.4. Effect of Oocytes on AMPK Protein Expression in Cumulus Cells

**Figure 4:**
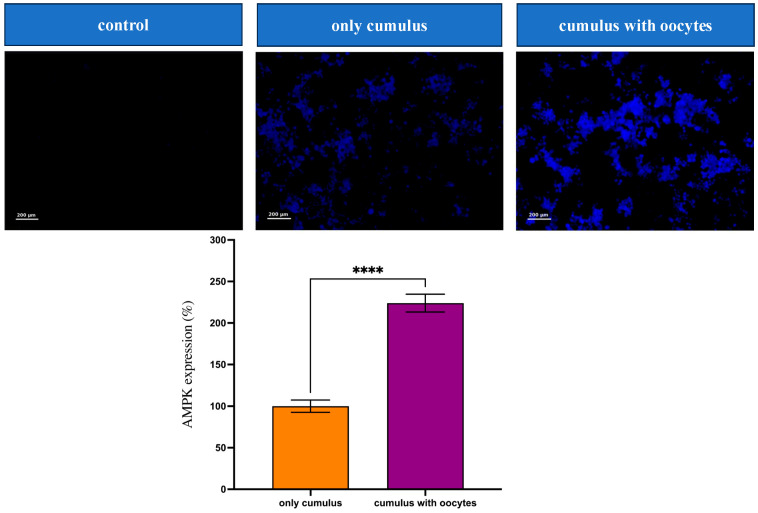
Immunocytochemical analysis of AMP-activated protein kinase (AMPK) expression (fluorescence representative images) in cumulus cells cultured under different conditions. (From left to right) negative control without primary antibody, cumulus without oocytes (group CCs-Oocytes), and cumulus co-cultured with oocytes (group CCs + Oocytes). Data are presented as mean ± SEM (**** *p* < 0.0001). AMPK, AMP-activated protein kinase.

AMPK protein expression in cumulus cells was significantly enhanced in the CC + Oocyte group compared to the only cumulus group. Immunocytochemical staining revealed that AMPK expression in the CC + Oocyte group was 228.0% of that observed in the only cumulus group (*p* < 0.0001). The Negative Control (No Primary Antibody) group exhibited no detectable fluorescence, confirming the specificity of the staining. These visualized and quantified results highlight the positive effect of oocyte–cumulus cell interactions on AMPK activation (Figure 4).

## 4. Discussion

This study demonstrates the critical role of cumulus cell–oocyte interactions in enhancing the oxidative and metabolic environment during in vitro culture of GV-stage oocytes. Co-culturing cumulus cells with oocytes significantly reduced intracellular ROS levels, enhanced AMPK protein expression in cumulus cells, and promoted the initiation of oocyte nuclear maturation, as reflected by a higher occurrence of germinal vesicle breakdown (GVBD). These findings underscore the intricate bidirectional communication between cumulus cells and oocytes, which is essential for optimizing in vitro culture conditions and advancing assisted reproductive technologies (ART).

### 4.1. Key Findings and Mechanistic Insights

Our results revealed that co-culture systems effectively mitigate oxidative stress, as evidenced by the significant reduction in ROS levels within cumulus cells. Oxidative stress, a major barrier to successful oocyte maturation, is known to impair cellular structures and functions critical for oocyte development. By co-culturing with oocytes, cumulus cells likely benefit from oocyte-secreted paracrine factors, such as growth differentiation factor 9 (GDF9) and bone morphogenetic protein 15 (BMP15), which enhance their antioxidant capacity [23,24,25,26]. The reduction in ROS levels suggests that oocyte-derived signaling actively regulates the oxidative balance within cumulus cells, providing a protective effect that supports the resumption of meiosis.

AMPK, a central regulator of cellular energy homeostasis, was significantly upregulated in cumulus cells co-cultured with oocytes. This activation aligns with its role in enhancing mitochondrial activity, boosting ATP production, and countering oxidative stress [27]. Elevated AMPK expression likely improves cumulus cell functionality, enabling them to better support oocyte metabolism and the initiation of nuclear maturation. The morphological and functional enhancements observed in cumulus cells co-cultured with oocytes further validate the importance of this interaction. Co-cultured cumulus cells exhibited greater cell–cell connectivity, pronounced extracellular matrix production, and robust cellular expansion compared to those cultured alone. These morphological improvements reflect the supportive role of oocyte-derived signals in promoting cumulus cell growth and differentiation.

### 4.2. Clinical Implications

These findings have significant implications for assisted reproductive technology (ART), particularly in optimizing IVM protocols for patients prone to oxidative stress, such as those with polycystic ovary syndrome (PCOS) or advanced maternal age [28]. By improving the oxidative and metabolic environment, co-culture systems can enhance the developmental potential of oocytes, leading to higher GVBD rates and improved fertilization outcomes. Additionally, reducing oxidative stress and enhancing AMPK activation in cumulus cells may help address the variability and inefficiency currently limiting the broader application of IVM [19].

The ability to modulate the oocyte microenvironment more effectively offers a promising strategy for improving ART success rates. These findings provide a strong rationale for incorporating co-culture systems into clinical practice to optimize IVM outcomes and improve overall fertility treatment efficacy.

From a clinical standpoint, the observed reduction in intracellular ROS and concomitant activation of AMPK signaling in co-cultured cumulus cells mirror the cellular responses seen in tissues under oxidative challenge, such as cardiac and hepatic ischemia or metabolic inflammation. These findings may help explain why AMPK activators—currently explored as therapeutic agents in diabetes, cardiovascular disease, and aging—could also play a pivotal role in enhancing oocyte competence and follicular resilience. Translating this cellular mechanism into assisted reproduction could enable pharmacological modulation of oxidative balance in the follicular microenvironment, improving oocyte maturation rates in high-risk patients.

In reproductive endocrinology, the present results provide a rationale for integrating oocyte–cumulus co-culture or AMPK-targeted preconditioning into existing IVM and IVF protocols. Such approaches may prove especially beneficial in conditions characterized by oxidative dysregulation, including PCOS, obesity-related infertility, and age-associated decline in oocyte quality. Furthermore, understanding oocyte-driven modulation of cumulus physiology could inform the design of artificial follicle systems and stem-cell–based ovarian tissue reconstruction, opening new perspectives in fertility preservation.

### 4.3. Study Limitations and Future Directions

Despite the promising findings, certain limitations must be acknowledged. Due to the rarity and precious nature of human oocytes, this study focused primarily on the analysis of GVBD occurrence as a proxy for nuclear maturation, as well as ROS fluorescence intensity and AMPK protein expression in cumulus cells. While these parameters provide valuable insights into the oocyte microenvironment [29], they do not directly assess the oxidative and metabolic states of the oocytes themselves.

Future studies should adopt advanced techniques such as single-cell transcriptomics, proteomics, and metabolomics to directly evaluate oocyte molecular profiles without compromising their viability. Expanding the sample size and incorporating additional markers of oxidative stress and energy metabolism across both cumulus cells and oocytes would provide more comprehensive insights and enhance the generalizability of the findings.

Also, further research should explore the temporal dynamics of ROS regulation and AMPK activation during co-culture [30,31]. Understanding how these processes correlate with critical stages of oocyte maturation, particularly the transition beyond GVBD to MI and MII stages, could inform the development of more precise interventions [32]. Additionally, investigating the roles of other key regulators, such as Sirtuins [33,34,35], mitochondrial dynamics, and autophagy-related pathways, could uncover new mechanisms underlying cumulus cell–oocyte interactions and their impact on IVM outcomes.

The bidirectional communication between oocytes and cumulus cells, as demonstrated in this study, exemplifies a self-regulating cellular partnership that maintains redox and energetic stability—principles central to both reproductive and general medicine. By highlighting the ability of developing oocytes to enhance cumulus cell viability and metabolic activity, these findings suggest that co-culture systems may serve as a physiological model for cell–cell cooperation under oxidative stress and could inform therapeutic interventions beyond reproduction. Clinically, incorporating such insights into ART protocols has the potential to improve outcomes for patients with compromised ovarian function, while conceptually, this research contributes to the broader understanding of how intercellular metabolic feedback can be harnessed to support tissue function and regeneration.

The positive influence of cumulus cells on co-cultured GV oocytes has been discussed for at least three decades. However, the aim of our study was fundamentally different: rather than examining how cumulus cells support oocytes, we investigated how oocytes influence cumulus cells under direct co-culture conditions. Cumulus cells are a somatic cell population with inherently variable viability, and their functional state can change markedly depending on the microenvironment. Our results demonstrate that the viability of cumulus cells increases significantly when they are co-cultured together with GV oocytes. This observation provides an important and previously underappreciated perspective on the bidirectional interaction between oocytes and their companion cells.

Our findings suggest that oocytes actively modulate the somatic compartment of the follicle, rather than being passive recipients of support, highlighting a reciprocal regulatory mechanism within the oocyte–cumulus complex.

## 5. Conclusions

The positive effect of in vitro co-culture reflects not only the support of oocyte maturation by cumulus cells but also the reciprocal enhancement in cumulus cell viability and function mediated by developing oocytes, underscoring the bidirectional nature of oocyte–cumulus communication.

## Figures and Tables

**Table 1:** Germinal vesicle breakdown (GVBD) rates in groups Oocytes-CCs and Oocytes + CCs.

Group	GV-Intact (n, %)	GVBD (n, %)	Total (n)	*p*-Value
Oocytes-CCs	9 (81.82%)	2 (18.18%)	11	
Oocytes + CCs	4 (33.33%)	8 (66.67%)	12	0.036 (*)
Total	13	10	23	

* *p* < 0.05 indicates statistical significance, analyzed by Fisher’s Exact Test.

## Data Availability

The datasets generated and/or analyzed during the current study are not publicly available due to patient confidentiality and the sensitive nature of the clinical data, but are available from the corresponding author upon reasonable request. Requestors will be required to submit a methodologically sound proposal and sign a data access agreement.
